# Medical Image Despeckling Using the Invertible Sparse Fuzzy Wavelet Transform with Nature-Inspired Minibatch Water Wave Swarm Optimization

**DOI:** 10.3390/diagnostics13182919

**Published:** 2023-09-12

**Authors:** Ahila Amarnath, Poongodi Manoharan, Buvaneswari Natarajan, Roobaea Alroobaea, Majed Alsafyani, Abdullah M. Baqasah, Ismail Keshta, Kaamran Raahemifar

**Affiliations:** 1 Indian Institute of Technology, Madras, Chennai 600036, Tamilnadu, India; 2 College of Science and Engineering, Hamad Bin Khalifa University, Doha P.O. Box 34110, Qatar; 3 Middlesex College, Edison, NJ 08818, USA; buvaneswariselvakumar@gmail.com; 4 Department of Computer Science, College of Computers and Information Technology, Taif University, P.O. Box 11099, Taif 21944, Saudi Arabia; 5 Department of Information Technology, College of Computers and Information Technology, Taif University, Taif 21974, Saudi Arabia; 6 Computer Science and Information Systems Department, College of Applied Sciences, AlMaarefa University, Riyadh 11597, Saudi Arabia; 7 Data Science and Artificial Intelligence Program, College of Information Sciences and Technology, Penn State University, State College, PA 16801, USA; 8 School of Optometry and Vision Science, Faculty of Science, University of Waterloo, 200 University, Waterloo, ON N2L3G1, Canada; 9 Faculty of Engineering, University of Waterloo, 200 University Ave W, Waterloo, ON N2L 3E9, Canada

**Keywords:** speckle noise, threshold, nature-inspired minibatch water wave swarm optimization, inveritible sparse fuzzy wavelet transform

## Abstract

Speckle noise is a pervasive problem in medical imaging, and conventional methods for despeckling often lead to loss of edge information due to smoothing. To address this issue, we propose a novel approach that combines a nature-inspired minibatch water wave swarm optimization (NIMWVSO) framework with an invertible sparse fuzzy wavelet transform (ISFWT) in the frequency domain. The ISFWT learns a non-linear redundant transform with a perfect reconstruction property that effectively removes noise while preserving structural and edge information in medical images. The resulting threshold is then used by the NIMWVSO to further reduce multiplicative speckle noise. Our approach was evaluated using the MSTAR dataset, and objective functions were based on two contrasting reference metrics, namely the peak signal-to-noise ratio (PSNR) and the mean structural similarity index metric (MSSIM). Our results show that the suggested approach outperforms modern filters and has significant generalization ability to unknown noise levels, while also being highly interpretable. By providing a new framework for despeckling medical images, our work has the potential to improve the accuracy and reliability of medical imaging diagnosis and treatment planning.

## 1. Introduction

Ultrasound imaging is widely used in the medical field. It may be used to scan the uterus, liver, kidneys, spleen, brain, heart, and other soft tissues. Because of its efficiency, quickness, and low price, scanning equipment is frequently utilized. In ultrasound pictures, speckle noise is a common problem that may be ascribed to the imaging method, which may be based on coherent waves such as acoustic or laser imaging. The sorts of noise that may be brought on by various outside factors and the transmission system itself include Gaussian, Poisson, blurred, speckle, and salt-and-pepper noise. The practice of removing background noise has grown in importance in medical imaging applications, and the most often used filters—the median, Gaussian, and Wiener filters—deliver the greatest results for each kind of noise. Picture smoothing, which often uses the best filters or the industry standard filters, is used in most image processing programs to remove noise. A denoising model’s capability is to remove noise from the image while still maintaining the edges. Unwanted noise may be eliminated using both linear and non-linear models. Despite their inability to adequately maintain image borders, linear models are often put to the test due to their speed. The histogram, size, and clarity of the MRI images are fed into filters, and depending on these inputs, the best filter is chosen. The area of image noise reduction benefits greatly from the usage of filtering techniques, which may be used to denoise a picture in a number of ways. It can be solved using a variety of algorithms. In order to remove noise without lowering the quality of the examined image, the best filtering techniques are utilized to identify noise with neighboring data.

This work presents a method for suppressing this kind of speckle noise and analyzes its performance. Mathematical morphology is the foundation of this method. It is an updated version of a previously developed algorithm. It is different from other algorithms since it does not rely on the histogram to determine an image’s threshold. It also employs a different approach for rebuilding the characteristics of the speckle’s size. In addition, it employs structuring components with an arbitrary structure that are similar to the forms of the speckles. In terms of both time complexity and output quality, this method improves upon its predecessor. The general contribution of the study may be summarized as follows:

- Implementation of the sparse fuzzy wavelet transform for obtaining the noisy threshold for analyzing the noisy areas in the image;
- Then, in order to accurately remove the speckle noise in the input image, the determined threshold was applied to NIMWVSO.

The remainder of this document is structured as follows. Section 2 discusses the earlier literature. The ISFWT and NIMWVSO for image denoising are described in Section 3. The experimental data examined throughout the procedure are presented in Section 4. Section 5 provides the general conclusion and suggestions for further development.

## 2. Related Work

Here are a few of the several reported techniques for speckle-noise reduction. The authors of [1] offer a method for eliminating spot noise from ultrasound images using kinetic gas molecule optimization (KGMO) and a Bayesian framework. Here, the window widths of the image patches are optimized by KGMO, and noise is removed from those windows by the Bayesian network. Five deep learning networks with varied network topologies are introduced by [2] to decrease the influence of speckle noise on ultrasound images. It also includes U-shaped networks with batch normalization and batch re-normalization layers, a denoising network based on residual connections, and a modified generative adversarial network. The autocoder network is built on a convolutional neural network (CNN) with dilated convolution layers. Researchers have created a hybrid strategy that makes use of Kuan and non-local means filters to reduce noise (as stated in [3]). They use a Kuan filter to first sharpen the edges, and then non-local techniques to eliminate the speckle noise. Furthermore, the performance of the proposed hybrid filter and its design parameters are optimized using a meta-heuristic known as the gray wolf optimizer. Ref. [4] proposes SORAMA (Semantic Object Region and Morphological Analysis), an excellent technique for analyzing semantic object regions. A scan is the first step, followed by a noise-reduction process. After that, image quality becomes better. The area of interest (ROI) for the picture is identified. The morphological processes of dilatation and erosion then blur the image. The polished image clearly shows the stone. If the stone is still hidden, noise reduction is performed once again, and the process is repeated until a smoothed image containing the stone is discovered. The use of a progressive feature fusion attention dense network (PFFADN) to eliminate speckle noise from OCT images is described in [5]. They first build up tightly connected dense blocks in the deep convolution network before connecting each shallowly generated feature map to the deep one to form a residual block. They use an attention mechanism to assist the network concentrate on the most crucial information while removing the rest. A uniform collection of feature maps that have been combined from all dense blocks are sent to the reconstruction output layer. There, a technique for removing the speckle noise that often shows up in ultrasound images is described [6,7]. These images are used in the medical industry to assist physicians in finding abnormalities and illnesses that are deeply buried inside a patient’s body. There have been a number of recommended filters for despeckling ultrasonic images, but it is still possible to enhance the quality of the denoised image to avoid false detection. This essay makes use of a bilateral filter that has been improved. The minimum mean brightness error bi-histogram equalization (MMBEBHE) approach was introduced to enhance the contrast of MRI images [8]. To reduce visual noise, this technique combines the Wiener and bilateral filters. The results of the study using the provided approach include results from speckle and Gaussian noise. In [9], the author describes the most popular convolutional neural network (CNN)-based despeckling techniques for ultrasound images, both in the transform domain and the spatial domain. Using transform domain methods such as wavelet, curvelet, and Bayes shrink has been successful in several studies. Deep-learning-based techniques, including DnCNN, ECNDNet, etc., enhance despeckling’s effectiveness. Here, image fusion is carried out by combining the speckle-noise-reduction (SNR) approach with the multi-modality image fusion method [10]. The SNR approach is used to eliminate background noise for enhanced medical image quality. The advantages and disadvantages of fusing many medical images into a single, meaningful image are also addressed. Additionally, the stationary wavelet transformation (SWT) technique is being studied as a more reliable approach of accomplishing the same goal. A novel fuzzy-logic-based non-local mean filter is introduced in [11] to model the speckle noise, recover the damaged image using fuzzy uncertainty modeling (FUM), smoothed by local statistic-based information while keeping the image characteristics for low and highly speckled ultrasound pictures. The presented denoising approach gathers local characteristics to use FUM in order to distinguish “similar and non-similar” non-local locations. By applying local statistical data to smooth these homogeneous zones, the fuzzy-logic-based noise-reduction approach is started. In this instance, an ultrasonic image is divided into subbands using the wavelet transform [12,13]. The approximation subband is modified using bilateral filtering, while the detail subbands are modified using thresholding and anisotropic diffusion. The smoothing and removal approaches covered in [14,15] are closely related to a number of processes (such as the identification of regions of interest) addressed in earlier studies that were examined here. Furthermore, defining this toolbox makes it simpler to conduct analyses and research with a more focused scope.

The classic methods are extensively covered in the first half of this research and include space, diffusion, and wavelet filters, to name just a few. The next section describes different state-of-the-art and hybrid models in the realm of speckle-noise filtering as well as contemporary, less well-known deep-learning-based machine learning techniques. A novel technique for reducing speckle noise in OCT volumes is presented in [16], which makes use of the matching en face representation to provide relatively speckle-free frontal parts of the retinal layers. The suggested method estimates the anatomical structures by resolving a constrained optimization problem that combines wavelet-domain sparsity and total variation (wavelet-TV) regularization in order to preserve the edges of retinal layers and lessen artifacts brought on by pure wavelet thresholding. To accomplish the goal function outlined in [17,18], we use a brand-new hybrid approach called the Randomized FireFly (FF) update in Lion Algorithm (RFU-LA), which includes components of both the Lion Algorithm (LA) and the FireFly Algorithm (FF). By taking the mean of images that have been routinely median-filtered using different kernel sizes, the hybrid median-mean filter (HM2F) proposed in [19] is a one-shot image processing method for decreasing speckle noise. Together, the median filter and mean technique may preserve up to 97 percentage of the original spatial resolution while producing a denoised image with reduced speckle contrast. The HM2F method is compared against several other well-known filtering techniques, including the classic median filter method, the 3D block-matching filter, the non-local means filter, the 2D windowed Fourier transform filter, and the Wiener filter, using a variety of speckle-distorted images. According to the authors of [20], there is a technique that uses a guided filter and speckle-reducing anisotropic diffusion (SRAD) to reduce the impact of speckle noise while maintaining sharp edges. First, speckle, a multiplicative noise, is removed using SRAD. After filtering, if there is still noise, it is added to using a logarithmic transformation. After the first filtering, any remaining noise in the image is removed using a guided filter. The final image is noise-free thanks to the exponential transform. The authors of [21,22,23,24,25] examine and summarize popular techniques for reducing speckle noise in ultrasonic images for the most part. We evaluate the different approaches via experiments, highlighting the distinctive contributions of each strategy to feature retention and denoising using quality assessments, texture analysis, and profile interpretation. Ref. [26] propose the method of computer-aided diagnostics that combines a wavelet neural network (WNN) and the grey wolf optimization (GWO) algorithm to find anomalies in breast ultrasound images [27]. The retrieved features have a substantial impact on the recognition rate of prior approaches, thus a new and improved CAD system based on a convolutional neural network (CNN) is developed to address them. It is capable of differentiating between those with normal control and those suffering from Alzheimer’s disease.

## 3. Problem Statement

Image quality is essential for analyzing or segmenting ultrasonic images because speckle hides small details. Recent studies have demonstrated that speckle reduction improves the expert’s visual perception while assessing ultrasound imaging of human organs. Processing ultrasonic images, which are used to offer essential diagnostic information about the human body, requires the removal of speckle noise. Speckle noise makes ultrasound images more difficult to visually analyze. The main objective of despeckling is to prevent losing tiny details or blurring the borders in ultrasonographic images. Speckle can be removed using a variety of approaches; however, noise reduction may be more difficult due to the higher threshold level. Speckle noise is a multiplicative noise, making it impossible to entirely remove it without altering the image’s boundaries and texture characteristics. This presents one of the technological obstacles in despeckling ultrasonic images. Traditional filtering methods frequently lead to a loss of resolution and fine characteristics as well, which might affect the accuracy of diagnostic data. Speckle noise has a non-Gaussian distribution and is strongly influenced by the imaging modality and tissue type, which makes it more challenging to find an ideal solution. Therefore, it is imperative to create an optimized method for speckle reduction that takes into account these particular difficulties while also maintaining image quality, resolution, and small details and edges. Additionally, quantitative metrics such as peak signal-to-noise ratio (PSNR), mean square error (MSE), structural similarity index (SSIM), and subjective visual quality assessment are frequently used to analyze the efficacy of despeckling algorithms. Therefore, when developing a despeckling algorithm for ultrasonic images, it is crucial to take both objective and subjective evaluation criteria into account.

## 4. Proposed Work

**Figure 1:**
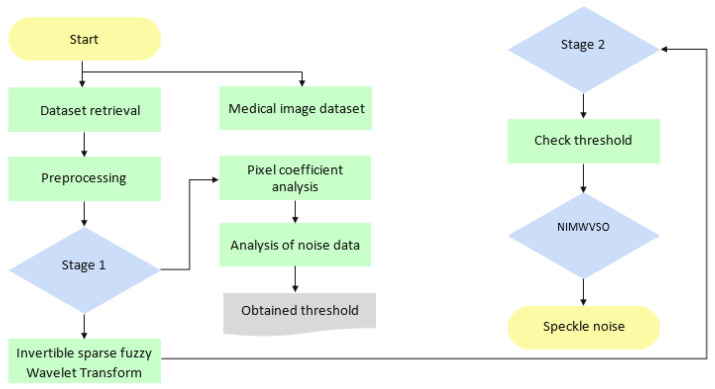
Schematic representation of the suggested methodology.

The ultrasound image quality is compromised by speckle, a kind of signal-dependent noise. Speckle noise has an additive impact. The diagnostic value of ultrasonic imaging is diminished as a result of this noise. Noise of this sort is an inevitable byproduct of medical ultrasound imaging. Therefore, anytime ultrasound imaging is utilized for medical imaging, speckle-noise reduction is a crucial pre-processing step. Figure 1 is a comprehensive representation of the proposed technique.

### 4.1. Data Source

**Figure 2:**
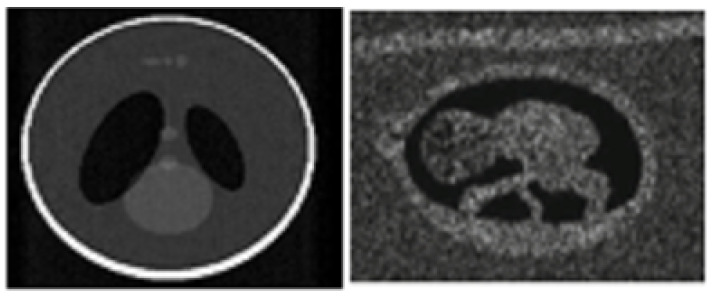
Sample input.

In these experiments, we just look at two different kinds of images. Figure 2 displays one kind of phantom picture, a 200 × 200 modified Shepp–Logan created using Matlab 2013’s phantom function (a). The noise variance was set to 0.1 in order to demonstrate the addition of speckle noise to this phantom picture. The second picture was a genuine ultrasound of the belly taken with a portable scanner (a SonoStar UBox-10 Ultrasound B Scanner equipped with a transabdominal convex probe operating at 3.5 MHz). Figure 2 shows an actual view of a fetal abdomen (b). Obstacles for the next phases include the phantom picture, the ambiguous limits of the abdomen, and the existence of speckle noise. Despeckling a picture is a crucial process that has to be accomplished in a way that does not compromise the image’s essential qualities. That is why we use a two-pronged approach in this case to eliminate the speckle noise.

### 4.2. System Model

Let $f_{ji}$ be the deteriorated, noisy picture,

(1) $$f_{ji}=a_{ji}*N_{ji}$$

where $a_{ji}$ and $N_{ji}$ are the multiplicative noise and the speckle-free picture pixel at position (j,i). $a_{ji}$ must be rescued from $f_{ji}$. Pixels’ coordinates in the picture space are indicated by the (j,i) subscripts.

The mathematical model of the speckle noise described in (2) demonstrates that the noise distribution in an ultrasound medical image is signal-dependent and multiplicative in nature, and is expressed as

(2) $$J^{noisy}\left(a\right)=J^{original}\left(a\right)\pm \left(a\right)$$

The original image is represented by $J^{original}\left(a\right)$. For each given pixel *a*, where an is an index, the observed picture, including noise, is denoted by $J^{noisy}\left(a\right)$ and ±(*a*) identifies a noise that is multiplicative in nature. The mathematical model is log transformed, leading to the following Equation (3):

(3) $$\log J^{noisy}\left(a\right)=\log\left(J^{original}\left(a\right)\right)+\log\pm \left(a\right))$$

The following formula describes the standard model of speckle noise:

(4) $$J^{noisy}\left(a\right)=J^{original}\left(a\right)+J^{original}{\left(a\right)}^{H}\pm \left(a\right)$$

where *H* is a constant associated with the zero-mean Gaussian distribution of the ultrasonic acquisition equipment. Setting r to 0.5 provides a decent approximation of the ultrasound data in the B-mode ultrasound picture research. When *H* is equal to 1, the model demonstrates that speckle noise is multiplicative.

The following equation is obtained by substituting a wavelet function into both sides of the expression.

(5) $$\ln f_{ji}=\ln a_{ji}+\ln N_{ji}$$

The wavelet transform is often used to shift medical images from the multiplicative speckle model to the additive noise model during processing and analysis. To estimate the medical picture devoid of speckles while still accounting for the logarithmic impact, an extra exponential threshold procedure is carried out.

### 4.3. ISFWT-Based Pixel Coefficient Analysis

The ISFWT detailed coefficients are determined using a thresholding method. Coefficients in the approximation are not thresholded in the same manner as the detailed coefficients are because they represent ’low-frequency’ terms that often comprise essential components of the signal and are less impacted by noise. The success of a threshold-based strategy depends on two main factors. The threshold value and the threshold function used are both crucial. Input images are segmented into $E\cup \forall^{K^{2}\times n_{e}}$ patches where each column represents a vectorized KK patch and $n_{e}$ may be thought of as the patch count. We offer a thresholding framework as an alternative to repeatedly picking low-rank patches $L:\forall^{\left(K^{2}\right)}\to \forall^{1}$ to estimate a scalar weight $C_{j}\cup \left[0,1\right]$ for each patch. With the use of soft selection, the suggested method may be trained to automatically give more weight to low-rank patches. The noise variance may be computed using the estimated weights and the patches by finding the lowest singular value in the weighted covariance matrix of the patch matrix:

(6) $$\pi^{2}=\frac{1}{\sum_{j=1}^{n_{e}}C_{j}}\pi min$$

where πmin determines the covariance matrix’s least singular value after weighting Ediag(C)ED with $=[1,\ldots ,n_{e}]D.$

The two most popular types of thresholding are hard threshold and soft threshold schemes. The hard threshold method disregards coefficients below a threshold value *D*, where *D* is determined by the variance of the noise. This method of decision making is sometimes referred to as the “keep or kill” approach.

(7) $$C_{\left(g,o\right)}=\left\{\begin{array}{cc} C_{\left(g,o\right)} & \mathrm{if}\;|C_{\left(g,o\right)}|\;\geq D\\ 0 & \mathrm{if}\;|C_{\left(g,o\right)}|\;<D \end{array}\right.$$

Using Equation (7) (above), we see that the soft threshold method reduces wavelet coefficients both above and below the threshold. The hard threshold produces results with harsher edges, while the soft threshold produces smoother results. A new exponential threshold is developed to deal with the drawbacks of both discontinuous and constant deviation in decision making, taking into consideration the benefits of the soft, hard, semi-soft, and Garrote threshold functions. The effectiveness of exponential threshold-based denoising is enhanced by this method. The exponential threshold function is defined mathematically as

(8) $$C_{\left(g,o\right)}=\left\{\begin{array}{cc} KNR\left(C_{\left(g,o\right)}\right)\left(\right|C_{g,o}|-1 & \mathrm{if}\;|C_{\left(g,o\right)}|\;\geq D\\ 0 & \mathrm{if}\;|C_{\left(g,o\right)}|\;<D \end{array}\right.$$

where $C_{\left(g,o\right)}$ may be easily manipulated using a shape parameter α. Since it is simply an ordinary integer, the scales may be set any way we want, and each signal will have its own value. It is important to carefully consider each element of Equation (9) since even little adjustments can significantly affect the amount of background noise. When the function’s α value approaches 0, it becomes a soft threshold. With ‘α’ at infinity, the function exhibits a severe cutoff. We may adjust the following settings in this exponential thresholding technique: ‘α’ and *D*. These adjusting factors are chosen by the optimization procedure. In order to maximize both smoothing and detail retention, we use two separate goal functions.

### 4.4. NIMWVSO-Based Noise Removal

Optimizing using NIMWVSO helps in determining the best settings for the models (‘α’ and *D*) of the exponential threshold function.

(9) $$\vartheta_{\left(g,o\right)}=\left\{\begin{array}{cc} KNR\left(C_{\left(g,o\right)}\right)\left\{(\right|C_{g,o}\left|-\frac{D}{exp^{3}\left[\alpha (\right|C_{g,o}\left|-D\right]/D}\right\} & \mathrm{if}\;|C_{\left(g,o\right)}|\;\geq D\\ 0 & \mathrm{if}\;|C_{\left(g,o\right)}|\;<D \end{array}\right.$$

That is, each particle $a_{j}$ is constructed such that $a_{j}=\left(D_{11},D_{12},......D_{ji}\right)$, where $D_{ji}$ is the *j*th particle’s *i*th tweak factor. Two tuning variables *t*, β and *d*, are selected for this suggested task. One such method is NIMWVS optimization, which has found usage in many different scientific and practical contexts with positive results. Here, any change in inertia weight will result in a shift in the wave particle’s position. The bigger the worth, the more accessible it is globally, yet the less feasible it is to mine locally. Results may be enhanced by using dynamic values rather than fixed ones. In this case, a more constrained radius for the search window, $R_{search}$, is convolved around a noisy $pixel_{j}$ dependent upon the found threshold. Utilizing the proposed based optimization statistics criterion, $pixel_{i}$ is similar to $pixel_{j}$. The radii of the local and global windows are similar, $O_{j}$ and $O_{i}$, centered at $pixel_{j}$ and $pixel_{I}$, respectively. Similarity between $pixel_{j}$ and all non-local neighboring $pixel_{I}$ uses a fitness function that is optimized for a certain geographical area to determine its value. The starting location is determined by the density of the swarming pixels. At this stage, the degree of similarity between windows is evaluated.For each non-local window $O_{i}$, an optimization-based approach is employed to determine the value of $O_{j}$. Looking for nearby, comparable non-local pixels may give a fair approximation of a noise-free pixel. Edge pixels often have extremely distinct values from the surrounding pixels and serve to visually separate two parts of a picture at first. Non-local neighbors, those that are not themselves part of an edge, are used to estimate noise-free pixels since they cannot distort those edges. These distorted edges may lead to incorrect diagnosis of illness using ultrasonic data. Due to the inherent uncertainty in noisy ultrasound images, it is challenging to locate similar patches or locations beyond the immediate vicinity of the central patch, $O_{j}$. To address these unknowns, tuning functions are created using an optimization-based approach. These functions incorporate the mean ratio and the variance ratio denoted as $G_{\theta }$ and $G_{\left(\tau^{2}\right)}$ respectively for the patches $O_{j}$ and $O_{i}$. These ratios are then utilized to determine the mutual resemblance grade. The similarity of the non-local window is then calculated as the sum of all of these similarities, $O_{i}$. With the information we have on the error threshold ratio, we may be able to pinpoint where the mistakes are being made. For regions with a greater membership degree, the local and non-local areas resemble one another more closely, but for regions with a lower membership degree, the $O_{i}$ belong to a different location; therefore, despeckling throws away those pixels. The similarity mechanism is based on the trapezoidal-shaped function of Equation (11).

(10) $$W\left(a:Z_{1},Z_{2},Z_{3},Z_{4}\right)=\left\{\begin{array}{cc} 0 & a\leq Z_{1}\\ \frac{a-z_{1}}{z_{1}-Z_{1}} & Z_{1}\leq a\leq Z_{2}\\ 1 & Z_{2}\leq a\leq Z_{3}\\ \frac{Z_{4}-a}{Z_{4}-Z_{3}} & Z_{3}\leq a\leq Z_{4}\\ 0 & Z_{4}\leq a \end{array}\right.$$

For the trapezoidal function, the input x-vector is denoted by an. The scalar factors are constants $Z_{1},Z_{2},Z_{3}$, and $Z_{4}$. Here, $Z_{1}$ and $Z_{4}$ denote the trapezoidal function’s bottom and top, respectively, whereas $Z_{2}$ and $Z_{3}$ denote its upper and lower bounds, respectively. The rigorous steps are used to derive these components and evaluate how similar $O_{j}$ and $O_{i}$ are as input windows. All comparable regions outside of the immediate area have now been counted. They are first restored after being smoothed using noise estimates based on optimal local statistics. Using the definition of the local linear minimum mean square error, we may generate the noise-free region w(a).

(11) $$J_{LLMMSE}\left(a\right)=P\left(J\left(a\right)\right)+\frac{\tau_{j}^{2}\left(a\right)}{\tau_{n}^{2}\left(a\right)}\left[n\left(a\right)-P\left(n\left(a\right)\right)\right]$$

where $J_{LLMMSE}\left(a\right)$ is the estimation of noise free image J(a); $\tau_{J}^{2}\left(a\right)$ and $\tau_{n}^{2}\left(a\right)$ noise in the picture *n*, and the J(a) variance (a). The expectations of the ideal picture J(a) and the input noisy patch n(a) are denoted by P(J(a)) and P(n(a)), correspondingly. These numbers are derived in the manner specified. Here, we use local statistics to compare non-locally similar patches and calculate the restored fitness value of a noisy pixel, j. Pixels that are visually identical also have the same statistical features. Euclidean distance are used to measure the proximity of two points on a map or at two different locations. The estimated noise level tends to cluster around low numbers rather than high ones. Zero-mean Gaussian distribution (θ=0) and an estimated noise variance $\left(\tau^{2}\right)$ are used to identify extra-regional participation $O_{i}$:

(12) $$\mathrm{weight}\left(\tau_{ji}\tau ,\theta \right)=p^{\frac{-{\left(t_{ji}-\theta \right)}^{2}}{2\tau^{2}}}$$

Euclidean distance $t_{ji}$ among the local region ‘*j*’ and non-local region ‘*i*’ is given by

(13) $$t_{ji}=∥O_{j}-O_{i}∥$$

The central pixel of patch $O_{j}$ has the value calculated by adding the values of all neighboring pixels that are not included in the local region. The predicted noise-free value is pixel *j* as a consequence of the fuzzy centroid technique employed in the defuzzification step, and this is written as

(14) $$pixel_{l}=\frac{1.0}{\sum_{g=1}^{N}weight_{g}}\sum_{g=1}^{N}\left(pixel_{g}Xweight_{g}\right)$$

The value of $pixel_{j}$ estimates the restored pixel’s value, where *N* is the number of non-locally comparable regions and local regions with similar values. $pixel_{g}$ is the value of the central pixel of window $O_{g}$, and $weight_{g}$ corresponds to the importance placed on the pixel with the highest degree of similarity as calculated by Equation (15).

“Case 1—Full Reference (FR) Measure”

Reference objective functions for the whole process include the peak-to-noise ratio (PSNR) and the mean structural similarity index metric (MSSSIM). Together, optimizing for these two aims produces a more distinct image overall while keeping the reaction smooth in otherwise similar regions. As the peak signal-to-noise ratio rises, so does the quality of the image following speckle reduction.

(15) $$PSNR\left(J_{D},J_{G}\right)=10\log_{10}\left[\frac{{\left(J_{G}\right)}_{peak}^{2}}{MSE}\right]$$

(16) $$MSE\left(J_{D},J_{G}\right)=\frac{1}{V_{n}}\sum_{j=1}^{V}\sum_{i=1}^{n}{\left(J_{g}\left(j,i\right)-J_{T}\left(j,i\right)\right)}^{2}$$

A further complete reference measure that places an emphasis on edge-preserving abilities is the mean structural similarity index. Two images’ degree of resemblance is quantified using the structural similarity index metric (SSIM). The MSSIM index may be between 0 and 1, with 1 indicating excellent edge preservation. A demonstration of MSSIM’s calculation is shown below.

(17) $$MSSIM\left(J_{T},J_{G}\right)=\frac{1}{V}\sum_{e=0}^{V-1}SSIM\left(J_{T},J_{G}\right)$$

(18) $$SSIM\left(J_{T},J_{G}\right)=\frac{\left(2\theta_{jt}\theta_{IG}+Z_{1}\right)\left(2\pi_{j_{T}I_{G}}+Z_{2}\right)}{\left(\theta_{JT}^{2}+\theta_{JG}^{2}+Z_{2}\right)\left(\pi_{JT}^{2}+\pi_{JG}^{2}+Z_{2}\right)}$$

where *V* is the number of the image’s local windows, JT and JG are the filtered and ground truth counterparts, and $Z_{1}$ and $Z_{2}$ are constants. The terms θ and π parallel the average and the deviation. The best solution is obtained by maximizing the objective functions I(J) (such as PSNR and MSSIM), as shown in Equation (20)

(19) J=arg(max(I(J)))

“Case 2—No Reference (NR) Measure”

The alpha–beta measure, which is derived from the ratio of the edge estimator and the despeckling evaluation index, is one example of a non-reference metric that may be used in MOPSO-tuned medical image despeckling (DEI). The estimator is then used to evaluate the filter’s noise-reduction capability, and the DEI of the Edge Around Region is then used to assess the filter’s edge-preserving abilities in this situation (EAR). Estimator examples include

(20) $$\beta_{\gamma \gamma }=\beta |\tau_{ENL}\left|+\left(1-\beta \right)\right|\tau_{\theta }|+\gamma$$

(21) $$\gamma =\frac{\sum_{j=1}^{S}\left({\left(J_{u}\right)}_{noisy}-{\left(\vec{J_{u}}\right)}_{noisy}\right)\left({\left(J_{u}\right)}_{ratio}-{\left(\vec{J_{u}}\right)}_{ratio}\right)}{\sum_{j=1}^{S}{\left({\left(J_{u}\right)}_{noisy}-{\left(\vec{J_{u}}\right)}_{noisy}\right)}^{2}\sum_{j=1}^{S}{\left({\left(J_{u}\right)}_{ratio}-{\left(\vec{J_{u}}\right)}_{ratio}\right)}^{2}}$$

where $J_{u}$ is the high-pass filtered image, $J_{u}$ is the average of the filtered image, $\tau_{E}NL$. The mean of the ratio image is the Equivalent Number of Looks (ENL), which is calculated by subtracting the noisy picture from the ratio image. Typically, the estimator’s values are between zero and one. A filter’s ability to preserve edges is measured using the despeckling evaluation index (DEI), calculated by dividing the standard deviation of a narrower neighborhood window by that of a broader one. The DEI value must be negative. If the data in the window has a bigger standard deviation, there will be more edges. So, a lower DEI is associated with better edge preservation ability.

(22) $$DEI=\frac{1}{V•n}\sum_{a,b}\frac{min(std\left(C_{e,U}^{v}\right)}{std(C_{a},b^{N})}$$

To improve edge maintenance and eliminate noise, the goal function here should have small values. Therefore, it is necessary to minimize the objective function, as shown in Equation (24).

(23) j=arg(min(I(J)))

Finally, the noises in the images are despeckled precisely.

## 5. Performance Analysis

The effectiveness of the approach that was recommended is analyzed in this section. Python was used as the operating system for the whole of the experiment. In order to show that the suggested network is superior to other conventional and well-known despeckling algorithms in terms of performance, the proposed network was compared with these other methods. Both actual ultrasounds and phantom images were used in the conducted experiments.

**Figure 3:**
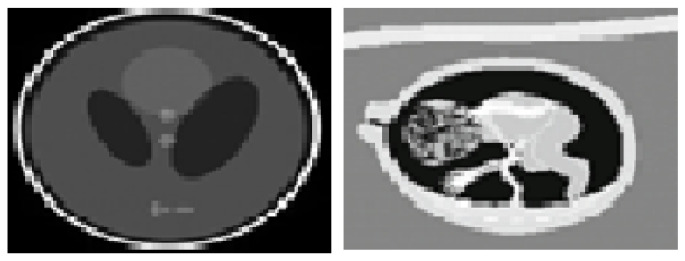
Denoised output.

**Figure 4:**
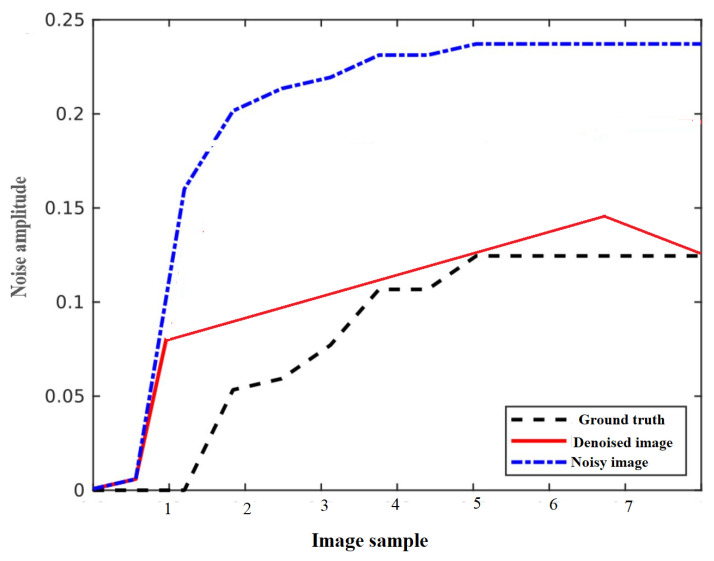
Image sample vs. noise amplitude.

Figure 3 shows the denoised output of the Shepp–Logan phantom which was built as a reference standard for use in evaluating the accuracy of head CT image reconstruction simulations. The ISFWT-NIMWVSO technique was used to denoise this phantom, as seen in the picture. Subsequently, the real-time fetal image was processed to remove noise, as seen in Figure 3 and the comparison of the input sample and noise amplitude shown in Figure 4.

In addition to this, we evaluated the estimate technique by applying it to phantom and fetal ultrasound images that had varying amounts of noise, and the results are shown in Table 1. We can observe that the suggested estimation technique has errors that are within acceptable ranges, since the estimated noise levels are somewhat higher than the actual low noise levels but slightly lower than the actual medium- and high-level estimates.

### Image Quality Evaluation Metrics

A novel algorithm for reducing speckle was recently shown. Several methods have been utilized to evaluate the efficiency of the proposed algorithm vs. the existing filters. Two common approaches to comparing results are quantitative assessments of image quality and qualitative evaluation, the latter of which is commonly carried out by the authors themselves. Significant effort has been made in recent years to establish objective metrics of image quality that correlate with assessments of how the quality is perceived. In order to evaluate filtering in relation to a speckle-free ideal reference, we use clinical and phantom images in addition to simulated ultrasonography as test data. However, it is difficult to tell how much enhancement was performed on an ultrasound image, and there is no universal way for doing so (Wang 2011). Quality evaluation metrics such as average difference (AD), Pratt’s figure of merit (FOM), root mean square error (RMSE), signal-to-noise ratio (SNR), peak signal-to-noise ratio (PSNR), maximum difference (MD), normalized absolute error (NAE), normalized cross-correlation (NK), structural content (SC), coefficient of correlation (CoC), universal quality index (UQI), and quality index (QI) were applied to the final images in this study (MSSIM). In addition to serving as an index, the final metric incorporates a visibility error map, which allows for the examination of areas in the distorted image where deviations occur between the original and distorted versions. Furthermore, we introduce a novel evaluation measure called the Speckle Reduction Score, which assesses the degree of improvement achieved. This new measure (SRS) mixes the edge preservation evaluation with a local similarity map.

- Signal-to-noise ratio (SNR): A common method for determining how well coherent imaging suppresses noise when that noise is multiplicative. This is achieved by comparing the intensity of the desired signal to that of the surrounding noise.
- Root mean square error (RMSE): This is calculated by squaring the intensity value difference between the original and denoised images and then dividing that number by the image’s size to obtain the root average.
- Peak signal-to-noise ratio (PSNR): This provides the image quality as a ratio of the original signal power to the signal power after denoising. A higher PSNR number indicates a better quality image.
- Pratt’s figure of merit (FOM) (Pratt 2007): In particular, this assesses how effectively an image retains its borders. The method used to generate a binary edge map has a significant impact on the FOM; higher PSNR values suggest better image quality. The Canny edge detector, which optimizes the FOM, is used for all of the speckle-reduction methods so that the results may be fairly compared. The FOM may take on any value between 0 and 1, with 1 representing optimal edge preservation.
- Normalized cross-correlation (NK): Its value (as a correlation-based measure of picture quality) is 1 for pairs of identical images.
- Average difference (AD): The average noise reduction is determined by comparing the original and denoised versions of a single pixel.
- Maximum difference (MD): Image denoising to the greatest possible degree.
- Normalized absolute error (NAE): This is a measurement of how effectively an image can predict its own mistakes.
- Coefficient of correlation (CoC): illustrates the linear relationship between the original and denoised photos and the direction in which that relationship runs.
- Quality index based on local variance (QILV) (Aja-Fernandez 2006): This theory relies on the observation that the local variance distribution of a picture encodes a great deal of the image’s structural information.
- Universal quality index (UQI) (Wang 2002): This is built on the idea that every distorted picture has three parts: a lack of correlation, distorted brightness, and distorted contrast.
- Laplacian mean square error (LMSE): Local contrast is an essential aspect of picture quality. Laplacian mean square error is the standard method for assessing picture local contrast.
- Mean structural similarity index map (MSSIM): We may use MSSIM to compare the lighting, contrast, and structure of two photos to see how closely they are alike (Wang, 2004). It may be used to discover comparable images for comparative purposes.
- Speckle Reduction Score (SRS): Several experiments have shown the usefulness of the MSSIM index, although there may be cases when the resulting quality measure does not agree with a subjective assessment based on visual data. A novel metric, the Speckle Reduction Score (SRS), is proposed and its computation is decomposed into two stages. The first stage is to determine the local similarity map, and the second is to combine the results from each map into a single value.

**Figure 5:**
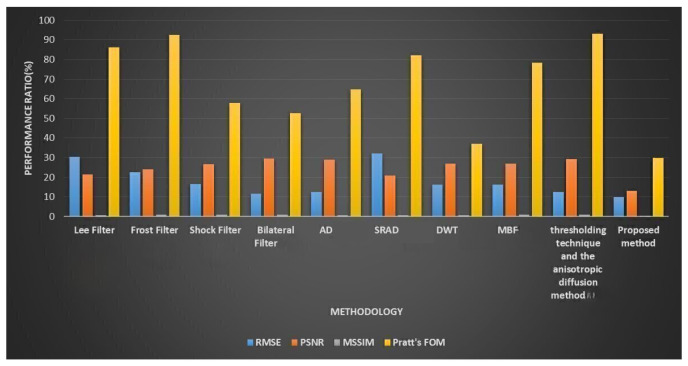
Performance analysis of the suggested mechanism.

Table 2 synthesizes the results of applying the improved speckle filter algorithm to the simulated picture by calculating a number of performance measures. Each metric’s optimal value is shown in bold. Figure 5 depicts performance analysis of the suggested mechanism.

**Figure 6:**
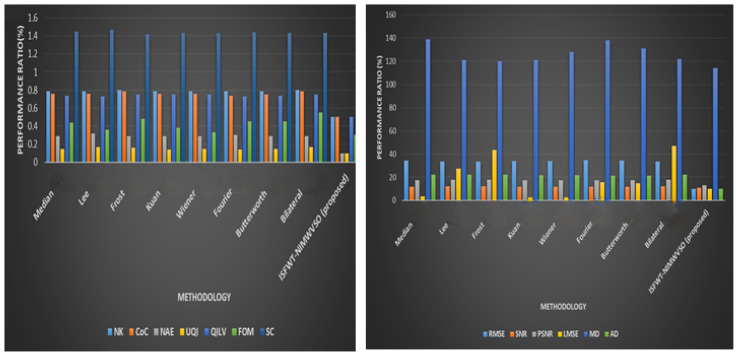
Performance evaluation.

**Figure 7:**
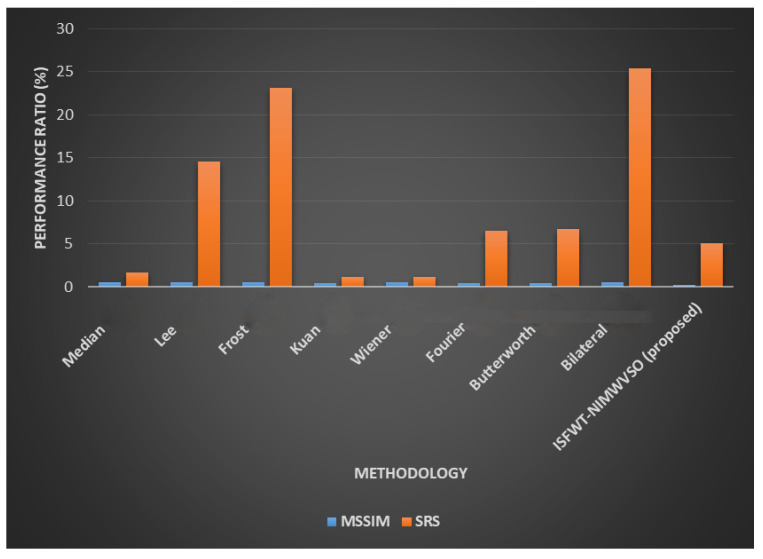
Performance evaluation.

Table 3 and Figure 6 compile the findings from several quality assessment techniques. Across the board, ISFWT-NIMWVSO filtering performs better than its competitors. As far as FOM is concerned, the suggested technique provides for more fulfilling results than do existing alternatives, enabling it to maintain its edge. Using the MSSIM, we can see that the suggested speckle filtering would maintain an appearance that is in line with how humans see things. The root mean squared error shows that the thresholding method employed in the multiresolution wavelet-based technique may keep the average error low all the way through the restoration procedure. By using optimization in place of a filter and a thresholding wavelet, our proposed method delivers the highest quantitative results. When the recommended method is implemented, the MSSIM index value dramatically rises. Thus, the proposed method yields an image that is very structurally similar to the standard. Since it is based on Pratt’s FOM, this method is more advantageous. So, this demonstrates that the method may continue to evolve and improve. The suggested method outperforms the current one since it increases the values of Pratt’s FOM, PSNR, and RMSE. The greatest noise reduction and edge retention is shown in Figure 6, thanks to the recommended method. The result of performance evaluation of the suggested mechanism is shown in Figure 7 and Table 4 compile the findings of performance metrics from existing and proposed method.

As shown by the result obtained, the suggested methodology outperforms other existing speckle filter methodologies in use.

## 6. Conclusions

In this study, we compare and contrast several widely used algorithms and methods for speckle-noise reduction from medical ultrasound images. A revised set of metrics for evaluating despeckling performance is provided. Research evaluating various methods of noise suppression in ultrasound images employed both the fetal image and a noise-free synthetic image of a phantom. The image was manipulated using Field II to add the typical noise seen in ultrasound images. Finally, we showed the proposed speckle smoothing algorithms in action by comparing them to the noise-free image. This study compiles an inventory of strategies for diminishing speckle, all of which are evaluated using qualitative metrics. Experimental findings on both simulated and clinical ultrasound pictures show quantitatively that the proposed method is better in terms of PSNR, SSIM, SRS, and coefficient of correlation. From what can be seen, the proposed approach outperforms the previous despeckling techniques in terms of reducing speckle noise and preserving clarity. In the future, we want to apply the proposed method to denoising other kinds of medical imaging, such as CT, MR, and PET scans, and to use deep learning to estimate the amount of speckle noise present in actual ultrasound images.

## Figures and Tables

**Table 1:** Estimate Technique.

Read Noise Levels	Mean of Estimated Noise Level	Mean Estimated Error
0.1	0.09	0.01
0.2	0.18	0.02
0.3	0.3	0
0.4	0.4	0
0.5	0.5	0

**Table 2:** Comparative Performance Analysis.

Method	RMSE	PSNR	MSSIM	Pratt’s FOM
Lee Filter [12]	30	21	0.783	86
Frost Filter [12]	23	24	0.88	93
Shock Filter [12]	17	27	0.877	58
Bilateral Filter [12]	12	30	0.882	53
AD [12]	13	29	0.78	65
SRAD [12]	32	21	0.757	82
DWT [12]	16	27	0.701	37
MBF [12]	16	27	0.861	78
Thresholding Technique and the Anisotropic Diffusion Method [12]	13	29	0.903	93
Proposed Method	10	13	0.2	30

**Table 3:** Proposed vs. existing comparative analysis.

Metrics	RMSE	SNR	PSNR	LMSE	MD	AD	NK
Median [21]	34.46	11.8	17.38	3.61	139	22.37	0.79
Lee [21]	33.28	12.03	17.74	27.43	121	22.31	0.79
Frost [21]	33.37	12.03	17.66	43.32	120	22.08	0.8
Kuan [21]	34.06	11.86	17.49	2.65	121	21.82	0.79
Wiener [21]	34.12	11.85	17.47	2.53	128	21.84	0.79
Fourier [21]	34.96	11.65	17.26	15.57	138	21.51	0.79
Butterworth [21]	34.29	11.8	17.43	14.76	131	21.43	0.79
Bilateral [21]	33.42	12.02	17.65	47.2	122	22.25	0.8
ISFWT-NIMWVSO (proposed)	10	11	13	10	114	10	0.5

**Table 4:** Proposed vs. existing comparative analysis.

Metrics	CoC	NAE	UQI	QILV	FOM	SC	MSSIM	SRS
Median [21]	0.76	0.29	0.15	0.74	0.44	1.45	0.47	1.68
Lee [21]	0.76	0.32	0.17	0.73	0.36	1.47	0.53	14.54
Frost [21]	0.79	0.29	0.16	0.75	0.48	1.42	0.53	23.09
Kuan [21]	0.76	0.29	0.14	0.75	0.38	1.43	0.44	1.17
Wiener [21]	0.76	0.29	0.15	0.75	0.33	1.43	0.47	1.18
Fourier [21]	0.74	0.3	0.14	0.73	0.45	1.44	0.42	6.47
Butterworth [21]	0.75	0.29	0.15	0.74	0.45	1.43	0.45	6.69
Bilateral [21]	0.79	0.29	0.17	0.75	0.55	1.43	0.54	25.38
ISFWT-NIMWVSO (proposed)	0.5	0.1	0.1	0.5	0.3	1.2	0.2	5

## Data Availability

The phantom picture used in our study, a modified Shepp-Logan image of size 200 × 200 was created using Matlab phantom function from the year 2013. The specific details and parameters of this modified Shepp-Logan phantom picture are available upon request.
